# Understanding the Research Landscape of Deep Learning in Biomedical Science: Scientometric Analysis

**DOI:** 10.2196/28114

**Published:** 2022-04-22

**Authors:** Seojin Nam, Donghun Kim, Woojin Jung, Yongjun Zhu

**Affiliations:** 1 Department of Library and Information Science Sungkyunkwan University Seoul Republic of Korea; 2 Department of Library and Information Science Yonsei University Seoul Republic of Korea

**Keywords:** deep learning, scientometric analysis, research publications, research landscape, research collaboration, knowledge diffusion

## Abstract

**Background:**

Advances in biomedical research using deep learning techniques have generated a large volume of related literature. However, there is a lack of scientometric studies that provide a bird’s-eye view of them. This absence has led to a partial and fragmented understanding of the field and its progress.

**Objective:**

This study aimed to gain a quantitative and qualitative understanding of the scientific domain by analyzing diverse bibliographic entities that represent the research landscape from multiple perspectives and levels of granularity.

**Methods:**

We searched and retrieved 978 deep learning studies in biomedicine from the PubMed database. A scientometric analysis was performed by analyzing the metadata, content of influential works, and cited references.

**Results:**

In the process, we identified the current leading fields, major research topics and techniques, knowledge diffusion, and research collaboration. There was a predominant focus on applying deep learning, especially convolutional neural networks, to radiology and medical imaging, whereas a few studies focused on protein or genome analysis. Radiology and medical imaging also appeared to be the most significant knowledge sources and an important field in knowledge diffusion, followed by computer science and electrical engineering. A coauthorship analysis revealed various collaborations among engineering-oriented and biomedicine-oriented clusters of disciplines.

**Conclusions:**

This study investigated the landscape of deep learning research in biomedicine and confirmed its interdisciplinary nature. Although it has been successful, we believe that there is a need for diverse applications in certain areas to further boost the contributions of deep learning in addressing biomedical research problems. We expect the results of this study to help researchers and communities better align their present and future work.

## Introduction

Deep learning is a class of machine learning techniques based on neural networks with multiple processing layers that learn representations of data [1,2]. Stemming from shallow neural networks, many deep learning architectures, such as convolutional neural networks (CNNs) and recurrent neural networks (RNNs), have been developed for various purposes [3]. The exponentially growing amount of data in many fields and recent advances in graphics processing units have further expedited research progress in the field. Deep learning has been actively applied to tasks, such as natural language processing (NLP), speech recognition, and computer vision, in various domains [1] and has shown promising results in diverse areas of biomedicine, including radiology [4], neurology [2], cardiology [5], cancer detection and diagnosis [6,7], radiotherapy [8], and genomics and structural biology [9-11]. Medical image analysis is a field that has actively used deep learning. For example, successful applications have been made in diagnosis [12], lesion classification or detection [13,14], organ and other substructure localization or segmentation [15,16], and image registration [17,18]. In addition, deep learning has also made an impact on predicting protein structures [19,20] and genomic sequencing [21-23] for biomarker development and drug design.

Despite the increasing number of published biomedical studies on deep learning techniques and applications, there has been a lack of scientometric studies that both qualitatively and quantitatively explore, analyze, and summarize the relevant studies to provide a bird’s-eye view of them. Previous studies have mostly provided qualitative reviews [2,9,10], and the few available bibliometric analyses were limited in their scope in that the researchers focused on a subarea such as public health [24] or a particular journal [25]. The absence of a coherent lens through which we can examine the field from multiple perspectives and levels of granularity leads to a partial and fragmented understanding of the field and its progress. To fill this gap, the aim of this study is to perform a scientometric analysis of metadata, content, and citations to investigate current leading fields, research topics, and techniques, as well as research collaboration and knowledge diffusion in deep learning research in biomedicine. Specifically, we intend to examine (1) biomedical journals that had frequently published deep learning studies and their coverage of research areas, (2) diseases and other biomedical entities that have been frequently studied with deep learning and their relationships, (3) major deep learning architectures in biomedicine and their specific applications, (4) research collaborations among disciplines and organizations, and (5) knowledge diffusion among different areas of study.

## Methods

### Data

Data were collected from PubMed, a citation and abstract database that includes biomedical literature from MEDLINE and other life science journals indexed with Medical Subject Heading (MeSH) terms [26]. MeSH is a hierarchically structured biomedical terminology with descriptors organized into 16 categories, with subcategories [27]. In this study, *deep learning [MeSH Major Topic]* was used as the query to search and download deep learning studies from PubMed. Limiting a MeSH term as a major topic increases the precision of retrieval so that only studies that are highly relevant to the topic are found [28]. As of January 1, 2020, a total of 978 PubMed records with publication years ranging from 2016 to 2020 have been retrieved using the National Center for Biotechnology Information Entrez application programming interface. Entrez is a data retrieval system that can be programmatically accessed through its Biopython module to search and export records from the National Center for Biotechnology Information’s databases, including PubMed [26,29]. The metadata of the collected bibliographic records included the PubMed identifier or PubMed ID, publication year, journal title and its electronic ISSN, MeSH descriptor terms, and author affiliations. We also downloaded the citation counts and references of each bibliographic record and considered data sources other than PubMed as well. We collected citation counts of the downloaded bibliographic records from Google Scholar (last updated on February 8, 2020) and the subject categories of their publishing journals from the Web of Science (WoS) Core Collection database using the electronic ISSN.

### Detailed Methods

#### Metadata Analysis

##### Journals

Journals are an important unit of analysis in scientometrics and have been used to understand specific research areas and disciplines [30]. In this study, biomedical journals that published deep learning studies were grouped using the WoS Core Collection subject categories and analyzed to identify widely studied research areas and disciplines.

##### MeSH Terms

Disease-related MeSH terms were analyzed to identify major diseases that have been studied using deep learning. We mapped descriptors to their corresponding numbers in MeSH Tree Structures to identify higher level concepts for descriptors that were too specific and ensured that all the descriptors had the same level of specificity. Ultimately, all descriptors were mapped to 6-digit tree numbers (C00.000), and terms with >1 tree number were separately counted for all the categories they belonged to. In addition, we visualized the co-occurrence network of major MeSH descriptors using VOSviewer (version 1.6.15) [31,32] and its clustering technique [33] to understand the relationships among the biomedical entities, as well as the clusters they form together.

##### Author Affiliations

We analyzed author affiliations to understand the major organizations and academic disciplines that were active in deep learning research. The affiliations of 4908 authors extracted from PubMed records were recorded in various formats and manually standardized. We manually reviewed the affiliations to extract organizations, universities, schools, colleges, and departments. For authors with multiple affiliations, we selected the first one listed, which is usually the primary. We also analyzed coauthorships to investigate research collaboration among organizations and disciplines. All the organizations were grouped into one of the following categories: universities, hospitals, companies, or research institutes and government agencies to understand research collaboration among different sectors. We classified medical schools under hospitals as they are normally affiliated with each other. In the category of research institutes or government agencies, we included nonprofit private organizations or foundations and research centers that do not belong to a university, hospital, or company. We extracted academic disciplines from the department section or the school or college section when department information was unavailable. As the extracted disciplines were not coherent with multiple levels and combinations, data were first cleaned with OpenRefine (originally developed by Metaweb then Google), an interactive data transformation tool for profiling and cleaning messy data [34], and then manually grouped based on WoS categories and MeSH Tree Structures according to the following rules. We treated interdisciplinary fields and fields with high occurrence as separate disciplines from their broader fields and aggregated multiple fields that frequently co-occurred under a single department name into a single discipline after reviewing their disciplinary similarities.

#### Content Analysis

We identified influential studies by examining their citation counts in PubMed and Google Scholar. Citation counts from Google Scholar were considered in addition to PubMed as Google Scholar’s substantial citation data encompasses WoS and Scopus citations [35]. After sorting the articles in descending order of citations, the 2 sources showed a Spearman rank correlation coefficient of 0.883. From the PubMed top 150 list (ie, citation count >7) and Google Scholar top 150 list (ie, citation count >36), we selected the top 109 articles. Among these, we selected the sources that met the criteria for applying or developing deep learning models as the subjects of analysis to understand the major deep learning architectures in biomedicine and their applications. Specifically, we analyzed the research topics of the studies, the data and architectures used for those purposes, and how the *black box* problem was addressed.

#### Cited Reference Analysis

We collected the references from downloaded articles that had PubMed IDs. Citations represent the diffusion of knowledge from cited to citing publications; therefore, analyzing the highly cited references in deep learning studies in biomedicine allows for the investigation of disciplines and studies that have greatly influenced the field. Toward this end, we visualized networks of knowledge diffusion among WoS subjects using Gephi (v0.9.2) [36] and examined metrics such as modularity, PageRank score, and weighted outdegree using modularity for community detection [37]. PageRank indicates the importance of a node by measuring the quantity and quality of its incoming edges [38], and weighted outdegree measures the number of outgoing edges of a node. We also reviewed the contents of the 10 most highly cited influential works.

## Results

### Metadata Analysis

#### Journals

On the basis of the data set, 315 biomedical journals have published deep learning studies, and Table 1 lists the top 10 journals selected based on publication size. Different WoS categories and MeSH terms are separated using semicolons.

From a total of 978 records, 96 (9.8%) were unindexed in the WoS Core Collection and were excluded, following which, an average of 2.02 (SD 1.19) categories were assigned per record. The top ten subject categories, which mostly pertained to (1) biomedicine, with 22.2% (196/882) articles published in *Radiology, Nuclear Medicine, and Medical Imaging* (along with *Engineering, Biomedical*: 121/882, 13.7%; *Mathematical and Computational Biology*: 107/882, 12.1%; *Biochemical Research Methods*: 103/882, 11.7%; *Biotechnology and Applied Microbiology*: 76/882, 8.6%; *Neurosciences*: 74/882, 8.4%); (2) computer science and engineering (*Computer Science, Interdisciplinary Applications*: 112/882, 12.7%; *Computer Science, Artificial Intelligence*: 75/882, 8.5%; *Engineering, Electrical and Electronic*: 75/882, 8.5%); or (3) *Multidisciplinary Sciences* (82/882, 9.3%).

**Table 1:** Top 10 journals with the highest record counts.

Journal title	Web of Science category	National Library of Medicine catalog Medical Subject Heading term	Publisher	Record count, n
*BMC^a^ Bioinformatics*	Biochemical Research Methods; Mathematical and Computational Biology; Biotechnology and Applied Microbiology	Computational Biology	BMC	38
*Scientific Reports*	Multidisciplinary Sciences	Natural Science Disciplines	Nature Research	37
*Neural Networks*	Neurosciences; Computer Science, Artificial Intelligence	Nerve Net; Nervous System	Elsevier	35
*Proceedings of the Annual International Conference of the IEEE^b^ Engineering in Medicine and Biology Society*	N/A^c^	Biomedical Engineering	IEEE	31
*IEEE Transactions on Medical Imaging*	Imaging Science and Photographic Technology; Engineering, Electrical and Electronic; Computer Science, Interdisciplinary Applications; Radiology, Nuclear Medicine, and Medical Imaging; Engineering, Biomedical	Electronics, Medical; Radiography	IEEE	30
*Sensors*	Chemistry, Analytical; Electrochemistry; Instruments and Instrumentation; Engineering, Electrical and Electronic	Biosensing Techniques	Multidisciplinary Digital Publishing Institute	26
*Bioinformatics*	Biochemical Research Methods; Mathematical and Computational Biology; Biotechnology and Applied Microbiology	Computational Biology; Genome	Oxford University Press	22
*Nature Methods*	Biochemical Research Methods	Biomedical Research/methods; Research Design	Nature Research	21
*Medical Physics*	Radiology, Nuclear Medicine, and Medical Imaging	Biophysics	American Association of Physicists in Medicine	20
*PloS one*	Multidisciplinary Sciences	Medicine; Science	Public Library of Science	20

^a^BMC: BioMed Central.

^b^IEEE: Institute of Electrical and Electronics Engineers.

^c^N/A: not applicable.

#### MeSH Terms

**Figure 1:**
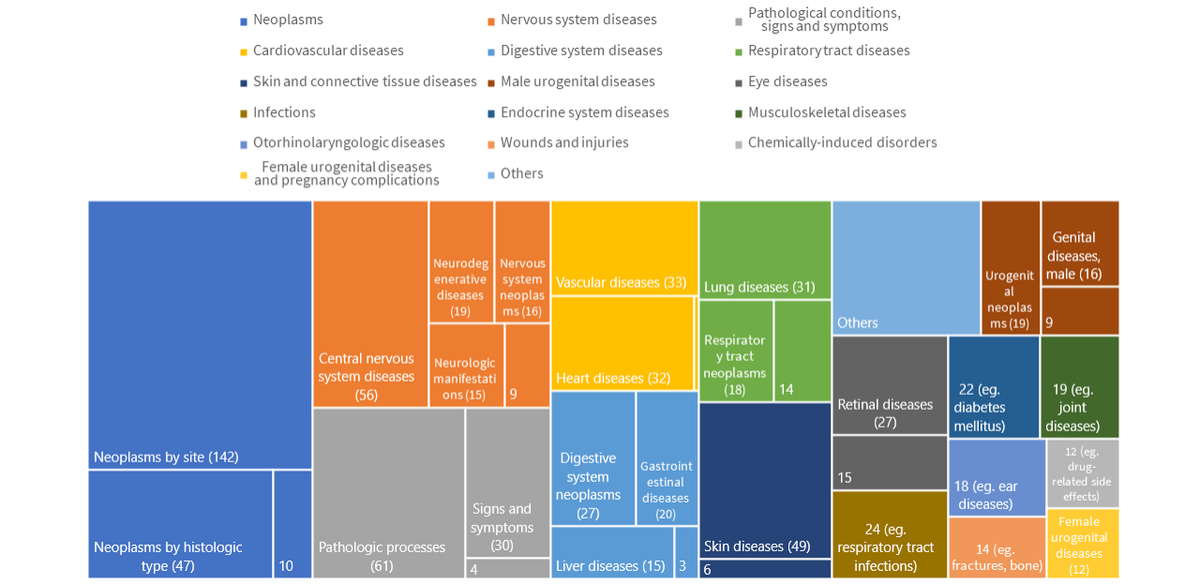
Disease-related Medical Subject Heading descriptors studied with deep learning.

For the main MeSH term or descriptor, an average of 9 (SD 4.21) terms was assigned to each record as subjects. Among them, we present in Figure 1 the diseases that were extracted from the *C* category. In the figure, the area size is proportional to the record count, and the terms are categorized by color. In addition, terms under >1 category were counted multiple times. For instance, the term *Digestive System Neoplasms* has two parents in MeSH Tree Structures, *Neoplasms* and *Digestive System Diseases*, and as such, we counted articles in this category under *Neoplasms* *by Site* as well as under *Digestive System Neoplasms*. Owing to the limited space, 7 categories whose total record counts were ≤10 (eg, *Congenital, Hereditary, and Neonatal Diseases and Abnormalities*; *Nutritional and Metabolic Diseases*; and *Stomatognathic Diseases*) were combined under the *Others* category, and individual diseases that had <10 record counts were summed up with each other in the same category to show only their total count (or with one of the diseases included as an example). In the process, we identified *Neoplasms* as the most frequently studied disease type, with a total of 199 studies.

**Figure 2:**
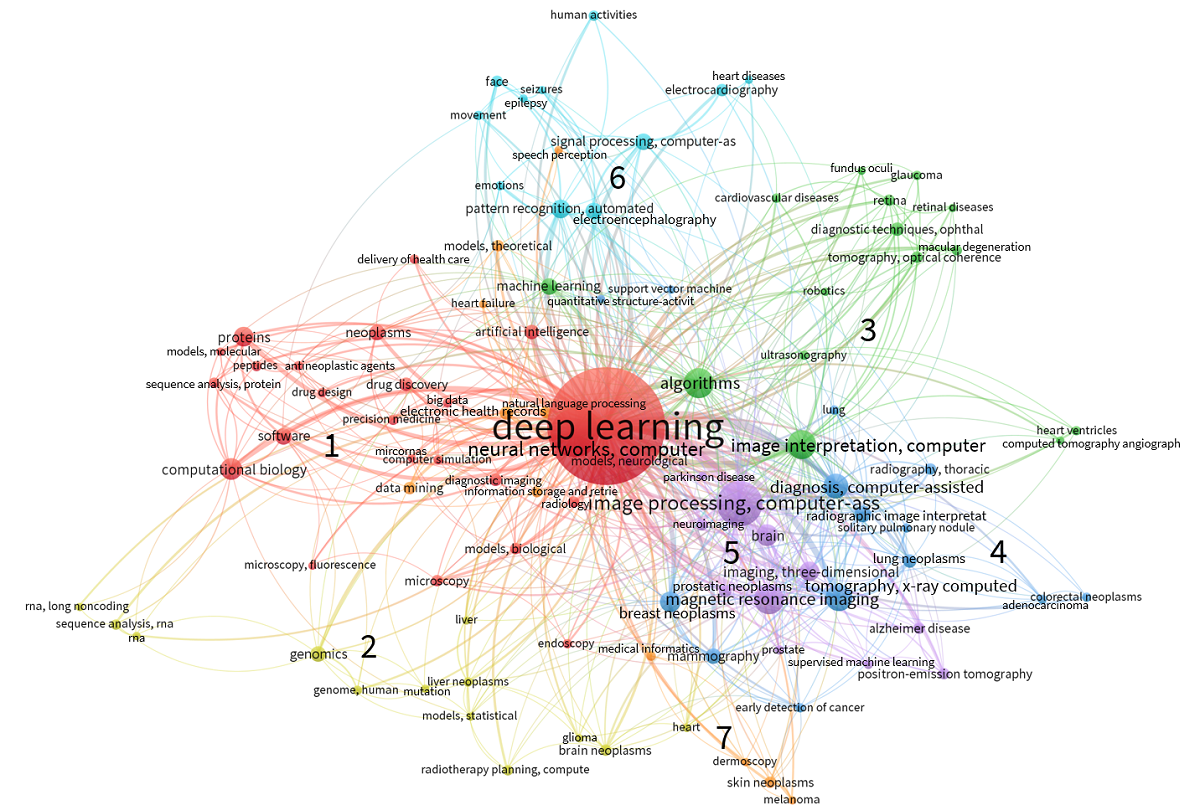
Co-occurrence network of the major Medical Subject Heading descriptors (number of nodes=100; number of edges=612; number of clusters=7).

We further constructed a co-occurrence network of the complete set of major MeSH descriptors assigned to the records to understand the relationships among the biomedical entities. To enhance legibility, we filtered out terms with <5 occurrences. Figure 2 presents the visualized network of nodes (100/966, 10.4% of the total terms) with 612 edges and 7 clusters. In the figure, the sizes of the nodes and edges are proportional to the number of occurrences, and the node color indicates the assigned cluster (although the term *deep learning* was considered nonexclusive to any cluster as it appeared in all records).

As depicted in Figure 2, each cluster comprised descriptors from two groups: (1) biomedical domains that deep learning was applied to, including body regions, related diseases, diagnostic imaging methods, and theoretical models, and (2) the purposes of deep learning and techniques used for the tasks, including diagnosis, analysis, and processing of biomedical data. In the first cluster, *computer neural networks* and *software* were studied for the purposes of *computational biology*, specifically *protein sequence analysis*, *drug discovery*, and *drug design*, to achieve *precision medicine*. These were relevant to the biomedical domains of (1) *proteins*, related visualization methods (*microscopy*), and *biological models*, and (2) *neoplasms*, related drugs (*antineoplastic agents*), and *diagnostic imaging* (*radiology*). In the second cluster, deep learning and *statistical models* were used for *RNA sequence analysis* and *computer-assisted radiotherapy planning* in relation to the domains of (1) *genomics*, *RNA*, and *mutation*, and (2) *brain neoplasms* and *liver neoplasms*. The third cluster comprised (1) heart structures (*heart ventricles*), *cardiovascular diseases*, and *ultrasonography* and (2) eye structures (*retina*), diseases (*glaucoma*), and *ophthalmological diagnostic techniques*. These had been studied for *computer-assisted image interpretation* using *machine learning* and deep learning *algorithms*. The biomedical domain group of the fourth cluster involved specific terms related to neoplasms such as type (*adenocarcinoma*), different regions (*breast neoplasms*, *lung neoplasms*, and *colorectal neoplasms*), and respective imaging methods (*mammography* and *X-ray computed tomography*) to which deep learning and *support vector machines* have been applied for the purpose of *computer-assisted radiographic image interpretation* and *computer-assisted diagnosis*. The fifth cluster included (1) *brain* disorders (*Alzheimer disease*), *neuroimaging*, and *neurological models*; (2) *prostatic neoplasms*; and (3) diagnostic *magnetic resonance imaging* and *3D imaging*. S*upervised machine learning* had been used for *computer-assisted image processing* of these data. In the sixth cluster, *automated pattern recognition* and *computer-assisted signal processing* were studied with (1) *human activities* (eg, *movement* and *face*), (2) abnormal brain activities (*epilepsy* and *seizures*) and monitoring methods (*electroencephalography*), and (3) *heart diseases* and *electrocardiography*. In the last cluster, *medical informatics*, specifically *data mining* and *NLP*, including *speech perception*, had been applied to (1) *electronic health records*, related *information storage and retrieval*, and *theoretical models* and (2) skin diseases (*skin neoplasms* and *melanoma*) and diagnostic *dermoscopy*.

#### Author Affiliations

**Figure 3:**
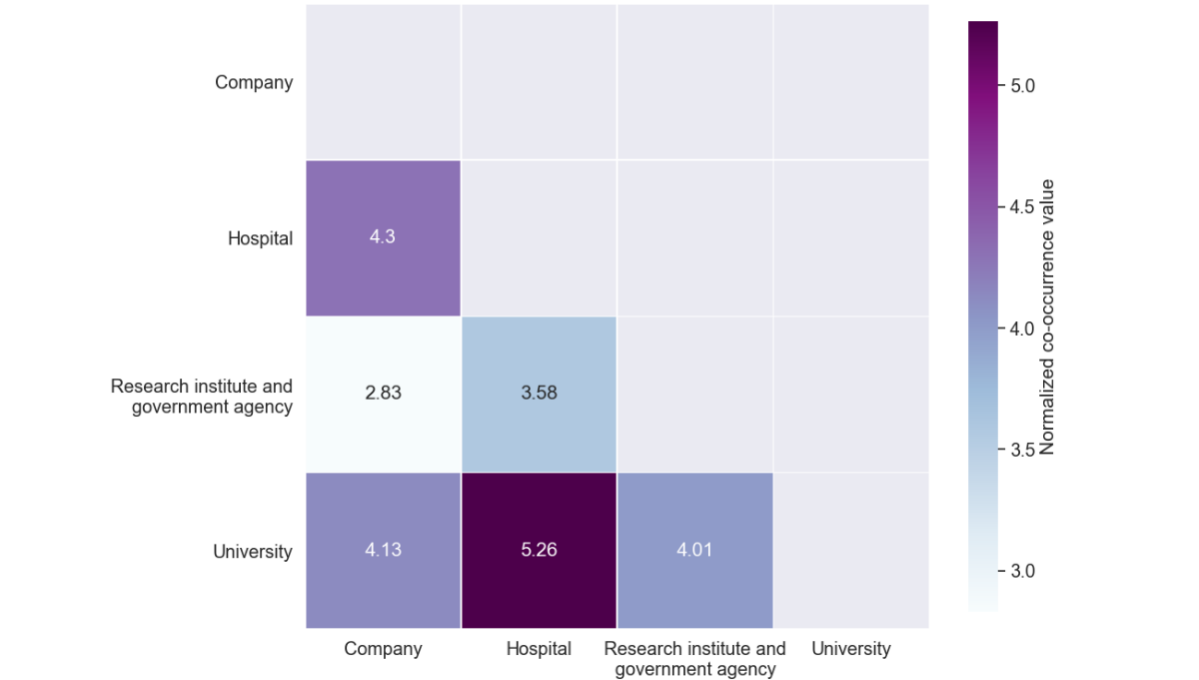
Collaboration of organization types.

To investigate research collaboration within the field, we analyzed paper-based coauthorships using author affiliations with different levels of granularity, including organization and academic disciplines. We extracted organizations from 98.7% (4844/4908) of the total affiliations and visualized the collaboration of different organization types. The top 10 organizations with the largest publication records included Harvard University (37/844, 4.4%), Chinese Academy of Sciences (21/844, 2.5%; eg, Institute of Computing Technology, Institute of Automation, and Shenzhen Institutes of Advanced Technology), Seoul National University (21/844, 2.5%), Stanford University (20/844, 2.4%), Sun Yat-sen University (14/844, 1.7%; eg, Zhongshan Ophthalmic Center and Collaborative Innovation Center of Cancer Medicine), University of California San Diego (14/844, 1.7%; eg, Institute for Genomic Medicine, Shiley Eye Institute, and Institute for Brain and Mind), University of California San Francisco (14/844, 1.7%), University of Michigan (14/844, 1.7%), Yonsei University (14/844, 1.7%), and the University of Texas Health Science Center at Houston (12/844, 1.4%). The extracted organizations were assigned to one of the following four categories according to their main purpose: universities, hospitals, companies, or research institutes and government agencies. Among these, universities participated in most papers (567/844, 67.2%), followed by hospitals (429/844, 50.8%), companies (139/844, 16.5%), and research institutes or government agencies (88/844, 10.4%). We used a co-occurrence matrix to visualize the degrees of organizational collaboration, with the co-occurrence values log normalized to compare the relative differences (Figure 3).

From Figure 3, we found that universities were the most active in collaborative research, particularly with hospitals, followed by companies and research institutes or government agencies. Hospitals also frequently collaborated with companies; however, research institutes or government agencies tended not to collaborate much as they published relatively fewer studies.

**Figure 4:**
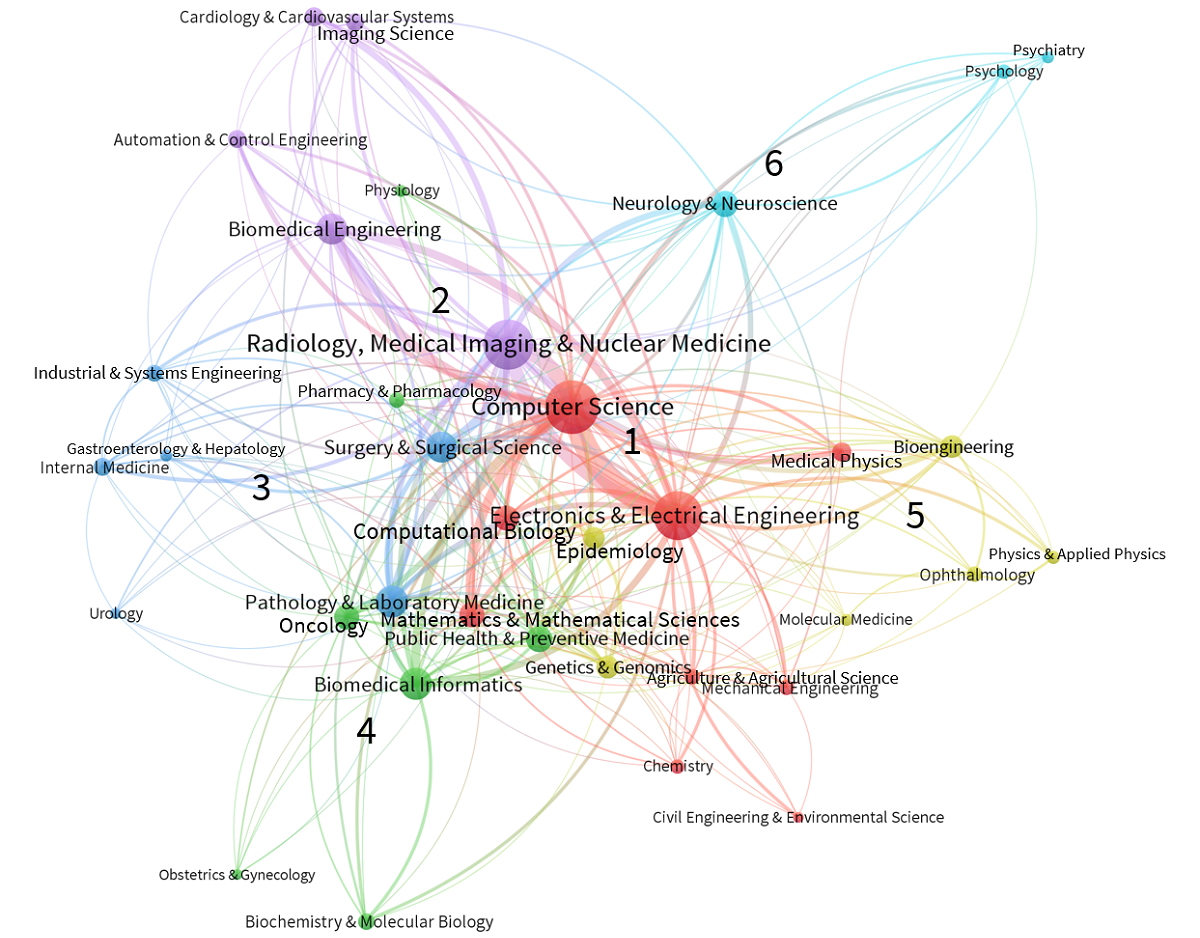
Collaboration network of academic disciplines (number of nodes=36; number of edges=267; number of clusters=6).

We also examined the collaborations among academic disciplines, which we could extract, as described in the *Methods* section, from 76.24% (3742/4908) of the total affiliations. Approximately half (ie, 386/756, 51.1%) of the papers were completed under disciplinary collaboration. Figure 4 depicts the network with 36 nodes (36/148, 24.3% of the total) and 267 edges after we filtered out disciplines with weighted degrees <10, representing the number of times one collaborated with the other disciplines. In the figure, the node and edge sizes are proportional to the weighted degree and link strength, respectively, and the node color indicates the assigned cluster.

As shown in the figure, the academic disciplines were assigned to 1 of 6 clusters, including 1 engineering-oriented cluster (cluster 1) and other clusters that encompassed biomedical fields. We specifically looked at the degree of collaboration between the biomedical and engineering disciplines. Figure 4 depicts that the most prominent collaboration was among *Radiology, Medical Imaging, and Nuclear Medicine*; *Computer Science*; and *Electronics and Electrical Engineering*. There were also strong links among *Computer Science* or *Electronics and Electrical Engineering* and *Biomedical Informatics*, *Biomedical Engineering*, and *Pathology and Laboratory Medicine*.

Among the top 10 disciplines in Figure 4, the following three had published the most papers and had the highest weighted degree and degree centralities: *Computer Science* (number of papers=195, weighted degree=193, and degree centrality=32); *Radiology, Medical Imaging, and Nuclear Medicine* (number of papers=168, weighted degree=166, and degree centrality=30); and *Electronics and Electrical Engineering* (number of papers=161, weighted degree=160, and degree centrality=32). Meanwhile, some disciplines had high weighted degrees compared with their publication counts, indicating their activeness in collaborative research. These included *Pathology and Laboratory Medicine* (5th in link strength vs 8th in publications) and *Public Health and Preventive Medicine* (9th in link strength vs 15th in publications). A counterexample was *Computational Biology*, which was 12th in link strength but 7th in publications.

### Content Analysis

#### Overview

We analyzed the content of influential studies that had made significant contributions to the field through the application or development of deep learning architectures. We identified these studies by examining the citation counts from PubMed and Google Scholar, assigning the 109 most-cited records to one of the following categories: (1) *review*, (2) *application* of existing deep learning architectures to certain biomedical domains (denoted by *A*), or (3) *development* of a novel deep learning model (denoted by *D*). Table 2 summarizes the 92 papers assigned to the application or development category according to their research topic in descending order of citation count.

**Table 2:** Top 92 studies with the highest citation count under the application or development category, according to the research topic.

Research topic and number			Task type		Data		Deep learning architectures
**(Diagnostic) image analysis**							
	A1 [39]	Classification		Retinal disease OCT^a^ and chest x-ray with pneumonia		Inception	
	A2 [40]	Segmentation and classification		Retinal disease OCT		U-net and CNN^b^	
	A3 [41]	Classification		Melanoma dermoscopic images		Inception	
	A4 [42]	Survival prediction		Brain glioblastoma MRI^c^		CNN_S	
	A6 [43]	Classification and segmentation		WSI^d^ of 13 cancer types		CNN with CAE^e^ and DeconvNet	
	D1 [44]	Segmentation		Brain MRI		ResNet^f^ based	
	A7 [45]	Prediction		Retinal fundus images with cardiovascular disease		Inception	
	D2 [46]	Tracking		Video of freely behaving animal		ResNet-based DeeperCut subset	
	A8 [47]	Classification		Colonoscopy video of colorectal polyps		Inception	
	A9 [48]	Classification		Lung cancer CT^g^		CNN	
	A10 [49]	Classification and segmentation		Retinal OCT with macular disease		Encoder-decoder CNN	
	D3 [50]	Segmentation		Brain glioma MRI		CNN based	
	D4 [51]	Binding affinities prediction		Protein-ligand complexes as voxel		SqueezeNet based	
	A11 [52]	Survival classification		Brain glioma MRI, functional MRI, and DTI^h^		CNN and mCNN^i^	
	A12 [53]	Classification		Fundus images with glaucomatous optic neuropathy		Inception	
	A13 [54]	Classification		Chest radiographs with pneumonia		ResNet and CheXNet	
	A14 [55]	Classification and segmentation		Critical head abnormality CT		ResNet, U-net, and DeepLab	
	A15 [56]	Classification		Brain glioma MRI		ResNet	
	D6 [57]	Classification		Thoracic disease radiographs		DenseNet based	
	A16 [58]	Classification and segmentation		Echocardiogram video with cardiac disease		VGGNet and U-net	
	A17 [59]	Classification		Brain positron emission tomography with Alzheimer		Inception	
	D7 [60]	Classification		Breast cancer histopathological images		CNN based	
	A18 [61]	Classification		Skin tumor images		ResNet	
	A19 [62]	Classification and prediction		Chest CT with chronic obstructive pulmonary disease and acute respiratory disease		CNN	
	A20 [63]	Segmentation		Brain MRI with autism spectrum disorder		FCNN^j^	
	D8 [16]	Segmentation		Fetal MRI and brain tumor MRI		Proposal network (P-Net) based	
	A21 [64]	Classification, prediction, and reconstruction		Natural movies and functional MRI of watching movies		AlexNet and De-CNN	
	D9 [65]	Detection and classification		Facial images with a genetic syndrome		CNN based	
	A22 [66]	Detection and segmentation		Microscopic images of cells		U-net	
	A23 [67]	Classification and localization		Breast cancer mammograms		Faster region-based CNN with VGGNet	
	A24 [68]	Segmentation and prediction		Lung cancer CT		Mask-RCNN, CNN with GoogLeNet and RetinaNet	
	A26 [69]	Classification		Lung cancer CT		CNN; fully connected NN; SAE^k^	
	A27 [70]	Survival classification		Lung cancer CT		CNN	
	A29 [71]	Prediction		Polar maps of myocardial perfusion imaging with CAD^l^		CNN	
	A30 [72]	Classification		Prostate cancer MRI		CNN	
	D12 [73]	Classification		Liver SWE^m^ with chronic hepatitis B		CNN based	
	D14 [74]	Segmentation		Liver cancer CT		DenseNet with U-net based	
	A31 [75]	Classification		Fundus images with macular degeneration		AlexNet, GoogLeNet, VGGNet, inception, ResNet, and inception-ResNet	
	A32 [76]	Classification		Bladder cancer CT		cuda-convnet	
	A34 [77]	Classification		Prostate cancer tissue microarray images		MobileNet	
	D19 [78]	Classification		Holographic microscopy of *Bacillus* species		CNN based	
	A36 [79]	Survival classification		Chest CT		CNN	
	D20 [80]	Classification and localization		Malignant lung nodule radiographs		ResNet based	
	A37 [81]	Classification		Shoulder radiographs with proximal humerus fracture		ResNet	
	A39 [82]	Classification		Facial images of hetero and homosexual		VGG-Face	
	A41 [83]	Segmentation and classification		CAD CT angiography		CNN and CAE	
	A42 [84]	Classification and localization		Radiographs with fracture		U-net	
	A43 [85]	Binding classification		Peptide major histocompatibility complex as image-like array		CNN	
	A44 [86]	Detection		Lung nodule CT		CNN	
	A45 [87]	Classification		Confocal endomicroscopy video of oral cancer		LeNet	
	A46 [88]	Classification		WSI of prostate, skin, and breast cancer		MIL^n^ with ResNet and RNN	
	D24 [89]	Tracking		Video of freely behaving animal		FCNN based	
	D25 [90]	Segmentation		Fundus images with glaucoma		U-net based	
	A47 [91]	Segmentation and classification		Cardiac disease cine MRI		U-net; M-Net; Dense U-net; SVF-Net; Grid-Net; Dilated CNN	
	D27 [92]	Classification		Knee abnormality MRI		AlexNet based	
	D28 [93]	Binding affinities prediction		Protein-ligand complexes as grid		CNN based	
	A50 [94]	Segmentation		Autosomal dominant polycystic kidney disease CT		FCNN with VGGNet	
	A51 [95]	Segmentation and classification		Knee cartilage lesion MRI		VGGNet	
	A52 [96]	Classification		Mammograms		ResNet	
	A54 [97]	Prediction		CAD CT angiography		FCNN	
	D31 [98]	Classification and localization		WSI of lymph nodes in metastatic breast cancer		Inception based	
	D35 [99]	Classification		Fluorescence microscopic images of cells		FFNN^o^ based	
	A56 [100]	Classification		Retinal fundus images with diabetic retinopathy and breast mass mammography		ResNet; GoogLeNet	
**Image processing**							
	A25 [101]	Artifact reduction		Brain and abdomen CT and radial MR^p^ data		U-net	
	A28 [102]	Resolution enhancement		Fluorescence microscopic images		GAN^q^ with U-net and CNN	
	D15 [103]	Dealiasing		Compressed sensing brain lesion and cardiac MRI		GAN with U-net and VGGNet based	
	D16 [104]	Resolution enhancement		Superresolution localization microscopic images		GAN with U-net–based pix2pix network modified	
	A33 [105]	Reconstruction		Brain and pelvic MRI and CT		GAN with FCNN and CNN	
	D18 [106]	Artifact reduction		CT		CNN based	
	A38 [107]	Reconstruction		Contrast-enhanced brain MRI		Encoder-decoder CNN	
	D22 [108]	Reconstruction		Brain MR fingerprinting data		FFNN based	
	D23 [109]	Resolution enhancement		Hi-C matrix of chromosomes		CNN based	
	A48 [110]	Resolution enhancement		Brain tumor MRI		U-net	
	D26 [111]	Reconstruction		Lung vessels CT		CNN based	
	D32 [112]	Resolution enhancement		Knee MRI		CNN based	
	D33 [113]	Reconstruction		CT		CNN based	
	D34 [18]	Registration		Cardiac cine MRI and chest CT		CNN based	
**Sequence analysis**							
	D17 [114]	Novel structures generation and property prediction		SMILES^r^		Stack-RNN^s^ with GRU^t^- and LSTM^u^ based	
	A40 [115]	Novel structures generation		SMILES		variational AE^v^; CNN- and RNN with GRU-based AAE^w^	
	D21 [116]	Gene expression (variant effects) prediction		Genomic sequence		CNN based	
	D30 [117]	Novel structures generation and classification		SMILES		GAN with differentiable neural computer and CNN based	
	A53 [118]	Novel structures generation		SMILES		LSTM	
	A57 [119]	Classification		Antimicrobial peptide sequence		CNN with LSTM	
**Sequence and image analysis**							
	D13 [120]	Contact prediction		Protein sequence to contact matrix		ResNet based	
**(Diagnostic) pattern analysis**							
	A5 [121]	Subtype identification (survival classification)		Multi-omics data from liver cancer		AE	
	D5 [122]	Phenotype prediction		Genotype		GoogLeNet and deeply supervised net based	
	D10 [123]	Survival prediction		Genomic profiles from cancer		FFNN based	
	D11 [124]	Drug synergies prediction		Gene expression profiles of cancer cell line and chemical descriptors of drugs		FFNN based	
	A35 [125]	NLP^x^ (classification)		Electronic health record with pediatric disease		Attention-based BLSTM^y^	
	A49 [126]	Binding classification		Protein sequence as matrix and drug molecular fingerprint		SAE	
	D29 [127]	Classification		Electrocardiogram signal		BLSTM based	
	A55 [128]	Classification		Polysomnogram signal		CNN	

^a^OCT: optical coherence tomography.

^b^CNN: convolutional neural network.

^c^MRI: magnetic resonance imaging.

^d^WSI: whole slide image.

^e^CAE: convolutional autoencoder.

^f^ResNet: residual networks.

^g^CT: computed tomography.

^h^DTI: diffusion tensor imaging.

^i^mCNN: multicolumn convolutional neural network.

^j^FCNN: fully convolutional neural network.

^k^SAE: stacked autoencoder.

^l^CAD: coronary artery disease.

^m^SWE: shear wave elastography.

^n^MIL: multiple instance learning.

^o^FFNN: feedforward neural network.

^p^MR: magnetic resonance.

^q^GAN: generative adversarial network.

^r^SMILES: simplified molecular input line-entry system.

^s^RNN: recurrent neural network.

^t^GRU: gated recurrent unit.

^u^LSTM: long short-term memory.

^v^AE: autoencoder.

^w^AAE: adversarial autoencoder.

^x^NLP: natural language processing.

^y^BLSTM: bidirectional long short-term memory.

#### Research Topics

In these studies, researchers applied or developed deep learning architectures mainly for the following purposes: image analysis, especially for diagnostic purposes, including the classification or prediction of diseases or survival, and the detection, localization, or segmentation of certain areas or abnormalities. These 3 tasks, which aim to identify the location of an object of interest, are different in that detection involves a single reference point, whereas localization involves an area identified through a bounding box, saliency map, or heatmap, segmentation involves a precise area with clear outlines identified through pixel-wise analysis. Meanwhile, in some studies, models for image analysis unrelated to diagnosis were proposed, such as classifying or segmenting cells in microscopic images and tracking moving animals in videos through pose estimation. Another major objective involved image processing for reconstructing or registering medical images. This included enhancing low-resolution images to high resolution, reconstructing images with different modalities or synthesized targets, reducing artifacts, dealiasing, and aligning medical images.

Meanwhile, several researchers used deep learning architectures to analyze molecules, proteins, and genomes for various purposes. These included drug design or discovery, specifically for generating novel molecular structures through sequence analysis and for predicting binding affinities through image analysis of complexes; understanding protein structure through image analysis of contact matrix; and predicting phenotypes, cancer survival, drug synergies, and genomic variant effects from genes or genomes. Finally, in some studies, deep learning was applied to the diagnostic classification of sequential data, including electrocardiogram or polysomnogram signals and electronic health records. In summary, in the reviewed literature, we identified a predominant focus on applying or developing deep learning models for image analysis regarding localization or diagnosis and image processing, with a few studies focusing on protein or genome analysis.

#### Deep Learning Architectures

Regarding the main architectures, most of them were predominantly CNNs and based on ≥1 CNN architecture such as a fully CNN (FCNN) and its variants, including U-net; residual neural network (ResNet) and its variants; GoogLeNet (Inception v1) or Inception and VGGNet and its variants; and other architectures. Meanwhile, a few researchers based their models on feedforward neural networks that were not CNNs, including autoencoders (AEs) such as convolutional AE and stacked AE. Others adapted RNNs, including (bidirectional) long short-term memory and gated recurrent unit. Furthermore, models that combined RNNs or AEs with CNNs were also proposed.

Content analysis of the reviewed literature showed that different deep learning architectures were used for different research tasks. Models for classification or prediction tasks using images were predominantly CNN based, with most being ResNet and GoogLeNet or Inception. ResNet with shortcut connections [129] and GoogLeNet or Inception with 1×1 convolutions, factorized convolutions, and regularizations [130,131] allow networks of increased depth and width by solving problems such as vanishing gradients and computational costs. These mostly analyzed medical images from magnetic resonance imaging or computed tomography, with cancer-related images often used as input data for diagnostic classification, in addition to image-like representations of protein complexes. Meanwhile, when applying these tasks to data other than images, such as genomic or gene expression profiles and protein sequence matrices, researchers used feedforward neural networks, including AEs, that enabled semi- or unsupervised learning and dimensionality reduction.

Image analysis for segmentation and image processing were achieved through CNN-based architectures as well, with most of them being FCNNs, especially U-net. FCNNs produce an input-sized pixel-wise prediction by replacing the last fully connected layers to convolution layers, making them advantageous for the abovementioned tasks [132], and U-net enhances these performances through long skip connections that concatenate feature maps from the encoder path to the decoder path [133]. In particular, for medical image processing tasks, a few researchers combined FCNNs (U-net) with other CNNs by adopting the generative adversarial network structure, which generates new instances that mimic the real data through an adversarial process between the generator and discriminator [134]. We found that images of the brain were often used as input data for these studies.

On the other hand, RNNs were applied to sequence analysis of the string representation of molecules (simplified molecular input line-entry system) and pattern analysis of sequential data such as signals. A few of these models, especially those generating novel molecular structures, combined RNNs with CNNs by adopting generative adversarial networks, including adversarial AE. In summary, the findings showed that the current deep learning models were predominantly CNN based, with most of them focusing on analyzing medical image data and different architectures that are preferred for the specific tasks.

Among these studies, Table 3 shows, in detail, the objectives and the proposed methods of the 35 studies with novel model development.

**Table 3:** Content analysis of the top 35 records in the development category.

Number	Development objectives	Methods (proposed model)
D1	Segment brain anatomical structures in 3D MRI^a^	Voxelwise Residual Network: trained through residual learning of volumetric feature representation and integrated with contextual information of different modalities and levels
D2	Estimate poses to track body parts in various animal behaviors	DeeperCut’s subset DeepLabCut: network fine-tuned on labeled body parts, with deconvolutional layers producing spatial probability densities to predict locations
D3	Predict isocitrate dehydrogenase 1 mutation in low-grade glioma with MRI radiomics analysis	Deep learning–based radiomics: segment tumor regions and directly extract radiomics image features from the last convolutional layer, which is encoded for feature selection and prediction
D4	Predict protein-ligand binding affinities represented by 3D descriptors	KDEEP: 3D network to predict binding affinity using voxel representation of protein-ligand complex with assigned property according to its atom type
D5	Predict phenotype from genotype through the biological hierarchy of cellular subsystems	DCell: visible neural network with structure following cellular subsystem hierarchy to predict cell growth phenotype and genetic interaction from genotype
D6	Classify and localize thoracic diseases in chest radiographs	DenseNet-based CheXNeXt: networks trained for each pathology to predict its presence and ensemble and localize indicative parts using class activation mappings
D7	Multi-classification of breast cancer from histopathological images	CSDCNN^b^: trained through end-to-end learning of hierarchical feature representation and optimized feature space distance between breast cancer classes
D8	Interactive segmentation of 2D and 3D medical images fine-tuned on a specific image	Bounding box and image-specific fine-tuning–based segmentation: trained for interactive image segmentation using bounding box and fine-tuned for specific image with or without scribble and weighted loss function
D9	Facial image analysis for identifying phenotypes of genetic syndromes	DeepGestalt: preprocessed for face detection and multiple regions and extracts phenotype to predict syndromes per region and aggregate probabilities for classification
D10	Predict cancer outcomes with genomic profiles through survival models optimization	SurvivalNet: deep survival model with high-dimensional genomic input and Bayesian hyperparameter optimization, interpreted using risk backpropagation
D11	Predict synergy effect of novel drug combinations for cancer treatment	DeepSynergy: predicts drug synergy value using cancer cell line gene expressions and chemical descriptors, which are normalized and combined through conic layers
D12	Classify liver fibrosis stages in chronic hepatitis B using radiomics of SWE^c^	DLRE^d^: predict the probability of liver fibrosis stages with quantitative radiomics approach through automatic feature extraction from SWE images
D13	Predict protein residue contact map at pixel level with protein features	RaptorX-Contact: combined networks to learn contact occurrence patterns from sequential and pairwise protein features to predict contacts simultaneously at pixel level
D14	Segment liver and tumor in abdominal CT^e^ scans	Hybrid Densely connected U-net: 2D and 3D networks to extract intra- and interslice features with volumetric contexts, optimized through hybrid feature fusion layer
D15	Reconstruct compressed sensing MRI to dealiased image	DAGAN^f^: conditional GAN^g^ stabilized by refinement learning, with the content loss combined adversarial loss incorporating frequency domain data
D16	Reconstruct sparse localization microscopy to superresolution image	Artificial Neural Network Accelerated–Photoactivated Localization Microscopy: trained with superresolution PALM^h^ as the target, compares reconstructed and target with loss functions containing conditional GAN
D17	Generate novel chemical compound design with desired properties	Reinforcement Learning for Structural Evolution: generate chemically feasible molecule as strings and predict its property, which is integrated with reinforcement learning to bias the design
D18	Reduce metal artifacts in reconstructed x-ray CT images	CNN^i^-based Metal Artifact Reduction: trained on images processed by other Metal Artifact Reduction methods and generates prior images through tissue processing and replaces metal-affected projections
D19	Predict *Bacillus* species to identify anthrax spores in single cell holographic images	HoloConvNet: trained with raw holographic images to directly recognize interspecies difference through representation learning using error backpropagation
D20	Classify and detect malignant pulmonary nodules in chest radiographs	Deep learning–based automatic detection: predict the probability of nodules per radiograph for classification and detect nodule location per nodule from activation value
D21	Predict tissue-specific gene expression and genomic variant effects on the expression	ExPecto: predict regulatory features from sequences and transform to spatial features and use linear models to predict tissue-specific expression and variant effects
D22	Reconstruct MRF^j^ to obtain tissue parameter maps	Deep reconstruction network: trained with a sparse dictionary that maps magnitude image to quantitative tissue parameter values for MRF reconstruction
D23	Generate high-resolution Hi-C interaction matrix of chromosomes from a low-resolution matrix	HiCPlus: predict high-resolution matrix through mapping regional interaction features of low-resolution to high-resolution submatrices using neighboring regions
D24	Estimate poses to track body parts of freely moving animals	LEAP^k^: videos preprocessed for egocentric alignment and body parts labeled using GUI^l^ and predicts each location by confidence maps with probability distributions
D25	Jointly segment optic disc and cup in fundus images for glaucoma screening	M-Net: multi-scale network for generating multi-label segmentation prediction maps of disc and cup regions using polar transformation
D26	Reconstruct limited-view PAT^m^ to high-resolution 3D images	Deep gradient descent: learned iterative image reconstruction, incorporated with gradient information of the data fit separately computed from training
D27	Predict classifications of and localize knee injuries from MRI	MRNet: networks trained for each diagnosis according to a series to predict its presence and combine probabilities for classification using logistic regression
D28	Predict binding affinities between 3D structures of protein-ligand complexes	Pafnucy: structure-based prediction using 3D grid representation of molecular complexes with different orientations as having same atom types
D29	Classify electrocardiogram signals based on wavelet transform	Deep bidirectional LSTM^n^ network–based wavelet sequences: generate decomposed frequency subbands of electrocardiogram signal as sequences by wavelet-based layer and use as input for classification
D30	Generate novel small molecule structures with possible biological activity	Reinforced Adversarial Neural Computer: combined with GAN and reinforcement learning, generates sequences matching the key feature distributions in the training molecule data
D31	Detect and localize breast cancer metastasis in digitized lymph nodes slides	LYmph Node Assistant: predict the likelihood of tumor in tissue area and generate a heat map for slides identifying likely areas
D32	Transform low-resolution thick slice knee MRI to high-resolution thin slices	DeepResolve: trained to compute residual images, which are added to low-resolution images to generate their high-resolution images
D33	Reconstruct sparse-view CT to suppress artifact and preserve feature	Learned Experts’ Assessment–Based Reconstruction Network: iterative reconstruction using previous compressive sensing methods, with fields of expert-applied regularization terms learned iteration dependently
D34	Unsupervised affine and deformable aligning of medical images	Deep Learning Image Registration: multistage registration network and unsupervised training to predict transformation parameters using image similarity and create warped moving images
D35	Classify subcellular localization patterns of proteins in microscopy images	Localization Cellular Annotation Tool: predict localization per cell for image-based classification of multi-localizing proteins, combined with gamer annotations for transfer learning

^a^MRI: magnetic resonance imaging.

^b^CSDCNN: class structure-based deep convolutional neural network.

^c^SWE: shear wave elastography.

^d^DLRE: deep learning radiomics of elastography.

^e^CT: computed tomography.

^f^DAGAN: Dealiasing Generative Adversarial Networks.

^g^GAN: generative adversarial network.

^h^PALM: photoactivated localization microscopy.

^i^CNN: convolutional neural network.

^j^MRF: magnetic resonance fingerprinting.

^k^LEAP: LEAP Estimates Animal Pose.

^l^GUI: graphical user interface.

^m^PAT: photoacoustic tomography.

^n^LSTM: long short-term memory.

#### Black Box Problem

In quite a few of the reviewed studies, the *black box* problem of deep learning was partly addressed, as researchers implemented various methods to improve model interpretability. To understand the prediction results of image analysis models, most used one of the following two techniques to visualize the important regions: (1) activation-based heatmaps [45,54,65,70], especially class activation maps [57,61,77,92], and saliency maps [59] and (2) occlusion testing [39,75,82,94]. For models analyzing data other than images, there were no generally accepted techniques for model interpretation, and researchers suggested some methods, including adopting an interpretable hierarchical structure such as the cellular subsystem [122] or anatomical division [125], using backpropagation [123], observing gate activations of cells in the neural network [114], or investigating how corrupted input data affect the prediction and how identical predictions are made for different inputs [93]. As such, various methods were found to be used to tackle this well-known limitation of deep learning.

### Cited Reference Analysis

On average, each examined deep learning study with at least one PubMed indexed citation (429/978, 43.9%) had 25.8 (SD 20.0) citations. These cited references comprised 9373 unique records that were cited 1.27 times on average (SD 2.16). Excluding the ones that were unindexed in the WoS Core Collection (8618/9373, 8.06% of the unique records), an average of 1.77 (SD 1.07) categories were assigned to a record. The top ten WoS categories, which were assigned to the greatest number of total cited references, pertained to the following three major groups: (1) biomedicine (*Radiology, Nuclear Medicine, and Medical Imaging*: 2025/11,033, 18.35%; *Biochemical Research Methods*: 1118/11,033, 10.13%; *Mathematical and Computational Biology*: 1066/11,033, 9.66%; *Biochemistry and Molecular Biology*: 1043/11,033, 9.45%; *Engineering, Biomedical*: 981/11,033, 8.89%; *Biotechnology and Applied Microbiology*: 916/11,033, 8.3%; *Neurosciences*: 844/11,033, 7.65%), (2) computer science and engineering (*Computer Science, Interdisciplinary Applications*: 1041/11,033, 9.44%; *Engineering, Electrical and Electronic*: 645/11,033, 5.85%), and (3) *Multidisciplinary Sciences* (with 1411/11,033, 12.79% records).

**Figure 5:**
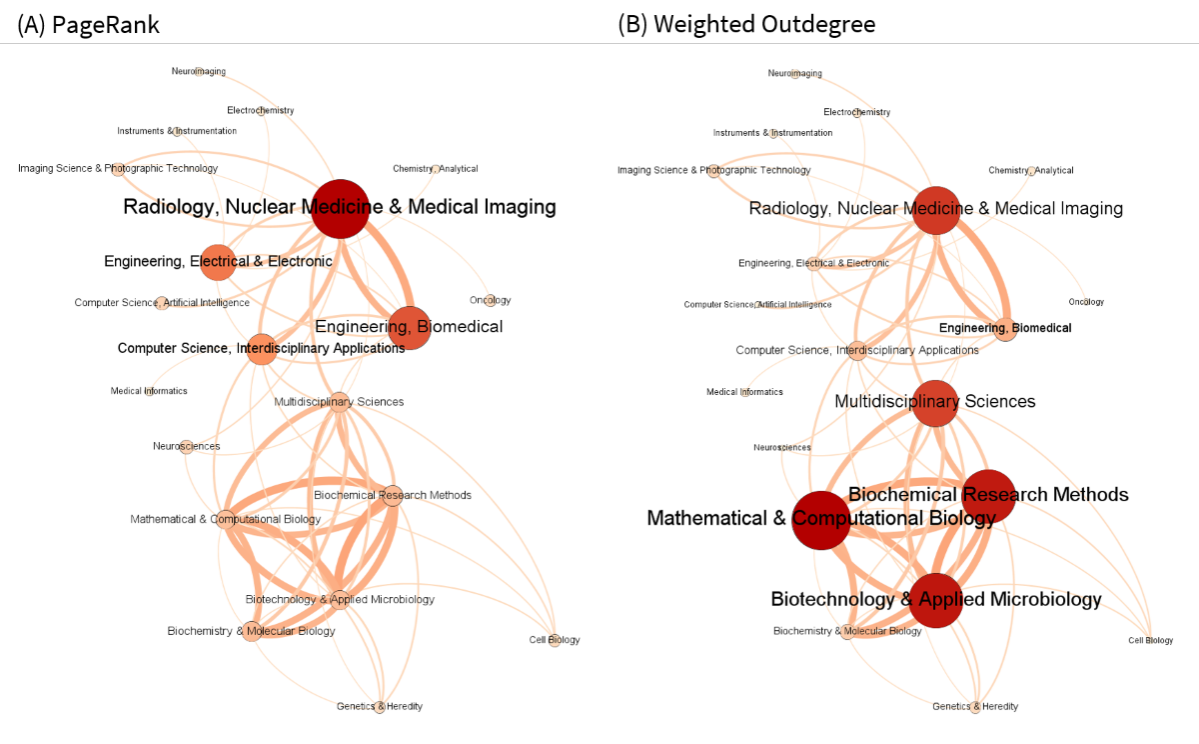
Citation network of the Web of Science subject categories assigned to the reviewed publications and their cited references according to (A) PageRank and (B) weighted outdegree (number of nodes=20; number of edges=59).

To understand the intellectual structure of how knowledge is transferred among different areas of study through citations, we visualized the citation network of WoS subject categories. In the directed citation network shown in Figure 5, the edges were directed clockwise with the source nodes as the WoS categories of the deep learning studies we examined and the target nodes as the WoS categories of the cited references from which knowledge was obtained. To enhance legibility, we filtered out categories with <100 weighted degrees, excluding self-loops, to form a network of 20 nodes (20/158, 12.7% of the total) and 59 edges (59/2380, 2.48% of the total). In the figure, the node color and size are proportional to the PageRank score (probability 0.85; ε=0.001; Figure 5A) and weighted-out degree (Figure 5B), and the edge size and color are proportional to the link strength. PageRank considers not only the quantity but also the quality of incoming edges, identifying important exporters for knowledge diffusion based on how often and by which fields a node is cited. On the other hand, the weighted outdegree measures outgoing edges and identifies major knowledge importers that frequently cite other fields.

As depicted in Figure 5A, categories with high PageRank scores mostly coincided with the frequently cited fields identified above and were grouped into two communities through modularity (upper half and lower half). The upper half region centered on Radiology, *Nuclear Medicine, and Medical Imaging*, which had the highest PageRank score (0.191) and proved to be a field with a significant influence on deep learning studies in biomedicine. Meanwhile, important knowledge exporters to this field included *Engineering, Biomedical* (0.134); *Engineering, Electrical and Electronic* (0.110); and *Computer Science, Interdisciplinary Applications* (0.091). The lower half region mainly comprised categories with comparable PageRank scores in which knowledge was frequently exchanged between one another, including *Biochemical Research Methods* (0.053), *Multidisciplinary Sciences* (0.053), *Biochemistry and Molecular Biology* (0.052), *Biotechnology and Applied Microbiology* (0.050), and *Mathematical and Computational Biology* (0.048). Specifically, in Figure 5B, *Mathematical and Computational Biology* (1992), *Biotechnology and Applied Microbiology* (1836), and *Biochemical Research Methods* (1807) were identified as major knowledge importers with the highest weighted outdegrees, whereas *Biochemistry and Molecular Biology* (344) had a relatively low weighted outdegree, indicating their role as a source of knowledge for these fields.

We analyzed the 10 most frequently cited studies to gain an in-depth understanding of the most influential works and assigned these papers to one of the three categories: review, application, or development. Review articles provided comprehensive overviews of the development and applications of deep learning [1,3], with 1 focusing on applications to medical image analysis [4]. We summarize the 7 application (denoted by *A*) or development (denoted by *D*) studies in Table 4.

In these studies, excluding the study by Hochreiter and Schmidhuber [135], whose research topic pertained to computer science, deep learning was used for diagnostic image analysis of various areas [12-14,136] and for sequence analysis of proteins [21] or genomes [22]. The main architectures implemented to achieve the different research objectives mostly comprised CNNs [12-14,136] or CNN-based novel models [21,22] and RNNs [135]. The findings indicated that these deep neural networks either outperformed previous methods or achieved a performance comparable with that of human experts.

**Table 4:** Content analysis matrix of the highly cited references in the application or development category.

Category	Citation count, n	Research topic: task type	Objectives	Methods (deep learning architectures)
A1 [12]	53	Diagnostic image analysis: classification	Apply CNN^a^ to classifying skin lesions from clinical images	Inception version 3 fine-tuned end to end with images; tested against dermatologists on 2 binary classifications
A2 [13]	51	Diagnostic image analysis: classification	Apply CNN to detecting referrable diabetic retinopathy on retinal fundus images	Inception version 3 trained and validated using 2 data sets of images graded by ophthalmologists
D1 [135]	34	Computer science	Develop a new gradient-based RNN^b^ to solve error backflow problems	LSTM^c^ achieved constant error flow through memory cells regulated by gate units; tested numerous times against other methods
D2 [21]	33	Sequence analysis: binding (variant effects) prediction	Propose a predictive model for sequence specificities of DNA- and RNA-binding proteins	CNN-based DeepBind trained fully automatically through parallel implementation to predict and visualize binding specificities and variation effects
A3 [14]	27	Diagnostic image analysis: classification	Evaluate factors of using CNNs for thoracoabdominal lymph node detection and interstitial lung disease classification	Compare performances of AlexNet, CifarNet, and GoogLeNet trained with transfer learning and different data set characteristics
D3 [22]	23	Sequence analysis: chromatin profiles (variant effects) prediction	Propose a model for predicting noncoding variant effects from genomic sequence	CNN-based DeepSEA trained for chromatin profile prediction to estimate variant effects with single nucleotide sensitivity and prioritize functional variants
A4 [136]	23	Diagnostic image analysis: classification	Evaluate CNNs for tuberculosis detection on chest radiographs	Compare performances of AlexNet and GoogLeNet and ensemble of 2 trained with transfer learning, augmented data set, and radiologist-augmented approach

^a^CNN: convolutional neural network.

^b^RNN: recurrent neural network.

^c^LSTM: long short-term memory.

## Discussion

### Principal Findings

With the increase in biomedical research using deep learning techniques, we aimed to gain a quantitative and qualitative understanding of the scientific domain, as reflected in the published literature. For this purpose, we conducted a scientometric analysis of deep learning studies in biomedicine.

Through the metadata and content analyses of bibliographic records, we identified the current leading fields and research topics, the most prominent being radiology and medical imaging. Other biomedical fields that have led this domain included biomedical engineering, mathematical and computational biology, and biochemical research methods. As part of interdisciplinary research, computer science and electrical engineering were important fields as well. The major research topics that were studied included computer-assisted image interpretation and diagnosis (which involved localizing or segmenting certain areas for classifying or predicting diseases), image processing such as medical image reconstruction or registration, and sequence analysis of proteins or RNA to understand protein structure and discover or design drugs. These topics were particularly prevalent in their application to neoplasms.

Furthermore, although deep learning techniques that had been proposed for these themes were predominantly CNN based, different architectures are preferred for different research tasks. The findings showed that CNN-based models mostly focused on analyzing medical image data, with RNN architectures for sequential data analysis and AEs for unsupervised dimensionality reduction yet to be actively explored. Other deep learning methods, such as deep belief networks [137,138], deep Q network [139], and dictionary learning [140], have also been applied to biomedical research but were excluded from the content analysis because of low citation count. As deep learning is a rapidly evolving field, future biomedical researchers should pay attention to the emerging trends and keep aware of state-of-the-art models for enhanced performance, such as transformer-based models, including bidirectional encoder representations from transformers for NLP [141]; wav2vec for speech recognition [142]; and the Swin transformer for computer vision tasks of image classification, segmentation, and object detection [143].

The findings from the analysis of the cited references revealed patterns of knowledge diffusion. In the analysis, radiology and medical imaging appeared to be the most significant knowledge source and an important field in the knowledge diffusion network. Relatedly, we identified knowledge exporters to this field, including biomedical engineering, electrical engineering, and computer science, as important, despite their relatively low citation counts. Furthermore, citation patterns revealed clique-like relationships among the four fields—biochemical research methods, biochemistry and molecular biology, biotechnology and applied microbiology, and mathematical and computational biology—with each being a source of knowledge and diffusion for the others.

Beyond knowledge diffusion, knowledge integration was also encouraged through collaboration among authors from different organizations and academic disciplines. Coauthorship analysis revealed active research collaboration between universities and hospitals and between hospitals and companies. Separately, we identified an engineering-oriented cluster and biomedicine-oriented clusters of disciplines, among which we observed a range of disciplinary collaborations, with the most prominent 2 between radiology and medical imaging and computer science and electrical engineering, which were the 3 disciplines that were most involved in publishing and collaboration. Meanwhile, pathology and public health showed a high collaborative research to publications ratio, whereas computational biology showed a low collaborative ratio.

### Limitations

This study has the following limitations that may have affected data analysis and interpretation. First, focusing only on published studies may have underrepresented the field. Second, publication data were only retrieved from PubMed; although PubMed is one of the largest databases for biomedical literature, other databases such as DataBase systems and Logic Programming may also include relevant studies. Third, the use of PubMed limited our data to biomedical journals and proceedings. Given that deep learning is an active research area in computer science, computer science conference articles are valuable sources of data that were not considered in this study. Finally, our current data retrieval strategy involved searching *deep learning* as the major MeSH term, which increased precision but may have omitted relevant studies that were not explicitly tagged as *deep learning*. We plan to expand our scope in future work to consider other bibliographic databases and search terms as well.

### Conclusions

In this study, we investigated the landscape of deep learning research in biomedicine and identified major research topics, influential works, knowledge diffusion, and research collaboration through scientometric analyses. The results showed a predominant focus on research applying deep learning techniques, especially CNNs, to radiology and medical imaging and confirmed the interdisciplinary nature of this domain, especially between engineering and biomedical fields. However, diverse biomedical applications of deep learning in the fields of genetics and genomics, medical informatics focusing on text or speech data, and signal processing of various activities (eg, brain, heart, and human) will further boost the contribution of deep learning in addressing biomedical research problems. As such, although deep learning research in biomedicine has been successful, we believe that there is a need for further exploration, and we expect the results of this study to help researchers and communities better align their present and future work.

## Abbreviations

AE: autoencoder
CNN: convolutional neural network
FCNN: fully convolutional neural network
MeSH: Medical Subject Heading
NLP: natural language processing
ResNet: residual neural network
RNN: recurrent neural network
WoS: Web of Science
